# Data-efficient machine learning methods in the ME-TIME study: Rationale and design of a longitudinal study to detect atrial fibrillation and heart failure from wearables

**DOI:** 10.1016/j.cvdhj.2023.09.001

**Published:** 2023-10-04

**Authors:** Arman Naseri, David Tax, Pim van der Harst, Marcel Reinders, Ivo van der Bilt

**Affiliations:** ∗ Department of Cardiology, Haga Teaching Hospital, The Hague, The Netherlands; † Pattern Recognition and Bioinformatics, Delft University of Technology, Delft, The Netherlands; ‡ Department of Cardiology, University Medical Center Utrecht, Utrecht, The Netherlands

**Keywords:** Wearables, mHealth, Atrial fibrillation, Heart failure, Smartwatch, Artificial intelligence, Machine learning, Multiple-instance learning, Self-supervised learning

## Abstract

**Background:**

Smartwatches enable continuous and noninvasive time series monitoring of cardiovascular biomarkers like heart rate (from photoplethysmograms), step counter, skin temperature, et cetera; as such, they have promise in assisting in early detection and prevention of cardiovascular disease. Although these biomarkers may not be directly useful to physicians, a machine learning (ML) model could find clinically relevant patterns. Unfortunately, ML models typically need supervised (ie, annotated) data, and labeling of large amounts of continuous data is very labor intensive. Therefore, ML methods that are data efficient, ie, needing a low number of labels, are required to detect potential clinical value in patterns found in wearable data.

**Objective:**

The primary study objective of the ME-TIME (Machine Learning Enabled Time Series Analysis in Medicine) study is to design an ML model that can detect atrial fibrillation (AF) and heart failure (HF) from wearable data in a data-efficient manner. To achieve this, self-supervised and weakly supervised learning techniques are used.

**Methods:**

Two hundred subjects (100 reference, 50 AF, and 50 HF) are being invited to participate in wearing a Fitbit fitness tracker for 3 months. Interested volunteers are sent a questionnaire to determine their health, in particular cardiovascular health. Volunteers without any (history of) serious illness are assigned to the reference group. Participants with AF and HF are recruited in the Haga teaching hospital in The Hague, The Netherlands.

**Results:**

Enrollment commenced on May 1, 2022, and as of the time of this report, 62 subjects have been included in the study. Preliminary analysis of the data reveals significant inter-subject variability. Notably, we identified heart rate recovery curves and time-delayed correlations between heart rate and step count as potential strong indicators for heart disease.

**Conclusion:**

Using self-supervised and multiple-instance learning techniques, we hypothesize that patterns specific to AF and HF can be found in continuous data obtained from smartwatches.

## Introduction

Cardiovascular disease is one of the leading causes of mortality globally^1^ and cardiovascular healthcare accounts for a large portion of global healthcare costs and expenses. Early detection and prevention will decrease the burden of cardiovascular disease and will therefore decrease mortality, morbidity, and costs. Cardiovascular monitoring using big data from wearables and machine learning can drastically increase the availability and efficiency of cardiovascular healthcare globally, at the fraction of the costs of conventional medical-grade devices.

Electrocardiogram (ECG) monitoring, such as Holters or implantable loop recorders, are the gold standard for monitoring of outpatients with known or suspected arrhythmias. However, they are burdensome, can only be used for a limited period of time, and are expensive. Implantable loop recorders are invasive and have to be manually activated and analyzed in the hospital. This severely limits the use of these devices for long-term home monitoring of patients, and they have suboptimal patient comfort. For patients with chronic cardiovascular diseases, such as atrial fibrillation and heart failure, this implies frequent hospital visits and sometimes even hospital admissions (associated with higher mortality) that can be prevented by continuous and adequate home monitoring.

With the widespread availability of reliable, consumer-grade wearables such as smartwatches, continuous monitoring of, for example, heart rate with photoplethysmography and step counting with accelerometers is possible. This monitoring is easy, patient friendly, and cost effective. Combining the power of large amounts of data (big data) and novel machine learning techniques, these time series can be used to detect and perhaps even predict cardiovascular disease, therefore improving patient care. There are some caveats, however, as not all wearables have the same characteristics and quality. Consequently, they have been used with moderate success.^2^^,^^3^ They also provide less informative diagnostic signals as compared to, for example, electrocardiography or other commonly used cardiologic diagnostic modalities. The challenge but also the strength of machine learning models is that they learn by example and therefore large amounts of data are needed for which the cardiovascular outcome (class label) has been determined. Typically, supervised learning is used, where each observation of the data has a class label. This must be done with ECGs, since photoplethysmography or derived signals are difficult to interpret by a clinician. This so-called labeling or annotating of signals by physicians is infeasible for the large amounts of (continuous) data required, and therefore semi-automated^4^^,^^5^ and fully automated^2^^,^^3^ ECG labeling systems^6^ have been developed. However, these still require a lot of manual labor from continuously monitored users.

Therefore, the objective of the ME-TIME is (early) detection and prevention of heart disease by leveraging time series data from smartwatches, a cloud-based infrastructure, and machine learning algorithms specifically designed to function effectively with minimal labeling efforts.

## Methods

### Study design and data collection

**Figure 1:**
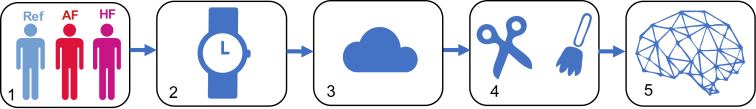
Data analysis pipeline for the ME-TIME study. Included participants (image 1) are given a smartwatch (image 2), which is connected to our data acquisition and storage platform (image 3). The resulting data are then preprocessed (image 4) and put into the data-efficient machine learning model (image 5). AF = atrial fibrillation group; HF = heart failure group; Ref = reference group.

ME-TIME (registered at ClinicalTrials.gov; ID: NCT05802563) is designed as an observational cohort study consisting of 3 data subject groups, as depicted in Figure 1. The first group consists of patients with systolic heart failure (HF group); the second group consists of patients with documented atrial fibrillation (AF group); and the third group, serving as a reference, consists of healthy volunteers. The rationale for creating distinct AF and HF groups comes from their unique pathophysiological characteristics. Consequently, heart rate patterns that are indicative of these diseases might also be different. The HF group consists of 50 study participants with systolic heart failure, defined as a left ventricular ejection fraction <35% without documented atrial fibrillation. The AF group consists of 50 patients with documented atrial fibrillation (paroxysmal, persistent, or permanent) without systolic heart failure. Ejection fractions will be assessed from echocardiograms that are made within 1 year of inclusion, and if this is not available an echocardiogram will be performed. The reference group consists of 100 participants without any prior medical history and without medication use. Potential study subjects that meet any of the following criteria will be excluded from participation in this study: age <18 years, age >85 years, recent pulmonary venous antrum isolation (<1 year), kidney or liver failure, known systemic active inflammatory disease, impaired mental state, inability to use a fitness tracker or mobile phone, impaired cognition, and inability to understand the study protocol.

Patients will be asked by their treating physician if they may be approached by an investigator to inform them about the study and potential participation. Healthy participants are recruited through local advertising. Anyone that is interested will then receive an information brochure and informed consent form. At least 2 days after the patient’s receipt of the brochure, the research team will call the patient to schedule an appointment. During this visit, the patient submits the signed consent form and will undergo an ECG and blood pressure measurement that will be analyzed by an experienced cardiologist (I.B.). Participants can use their own Fitbit and are otherwise provided with a Fitbit Inspire 2 or Fitbit Charge 5 smartwatch. The device type is assigned to a participant at random to prevent device sampling bias. This will also help to investigate the effect of device type on the performance of the final model. A Fitbit account will be created for all participants which will be connected to a custom-built data platform using the Google Cloud Platform. Our platform features a data portal for research staff to easily register or deregister participants by authorizing a connection to their Fitbit data. Data are extracted daily from Fitbits until the observation period ends and can be analyzed either in the cloud or locally.

All participants will be asked to fill out a survey regarding their health. All participants are monitored for a period of 3 months. After written consent from the 200 subjects, heart rate, step counter, and sleep time series data are extracted from the data platform. Clinical metadata such as age, height, weight, blood pressure at baseline, health survey, and medication use are saved in the Castor (Ciwit BV, Amsterdam, The Netherlands) electronic database.

### Data privacy

After performing a thorough data protection impact assessment, the local hospital security information and privacy officers granted permission to perform the study. This was also validated by the ethics review board. The data protection impact assessment describes a data management plan conforming to the European General Data Protection Regulation. To protect the data privacy of the participants, all data are pseudonymized. Only the researchers have access to the sensor data, and only the Principal Investigator has access to personal information of participants (ie, names, contact information, etc). They have all signed processing agreements. Second, the Google servers storing the data are only located within the Netherlands; hence the data does not leave the country, therefore conforming to Dutch law. This is done to have a clear data infrastructure both legally and technically to explain to participants.

### Data characteristics and preparation

The data first undergoes a process involving resampling and artifact removal. In our experience with Fitbit smartwatches, the heart rate is nonuniformly sampled, with a prevalent rate of 0.2 Hz. Therefore, the heart rate is resampled to once per 5 seconds. The step counter is sampled once per minute.

Artifacts involving samples with numerous consecutive constant values are removed, if more than 12 consecutive constant values (equivalent to 1 minute of heart rate samples) are detected. This 1-minute threshold was chosen based on visual inspection, which revealed that heart rate patterns typically occur in the order of minutes, often spanning 10–20 minutes. For sequences with fewer than 12 consecutive missing values, linear interpolation is applied. From the cleaned time series, smaller segments, denoted as windows, are extracted and employed as input for a machine learning model. This process involves a sliding window and windows containing time gaps are excluded. Windows have 2 design considerations: the window size, which determines the number of samples within a window and defines its dimensionality, and the stride, which establishes the step size dictating the shift between windows.

**Figure 2:**
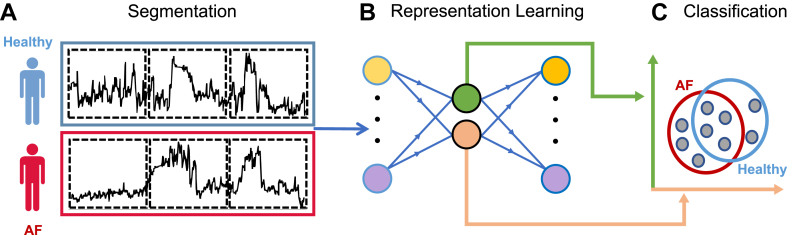
Pipeline for the study’s proposed approach. **a:** Segmentation of the time series of each subject (1 healthy and 2 atrial fibrillation) using a sliding window. Only the label of the entire subject is available, instead of each individual window. **b:** The windows are inputs to an autoencoder and are compressed to a smaller (2-dimensional for illustrative purposes) representation. **c:** The compressed representation is used to train a multiple-instance classifier that can distinguish between healthy, atrial fibrillation, or heart failure). AF = atrial fibrillation.

Although the cardiovascular condition of each subject is known, it is unknown in which specific windows these conditions manifest themselves. This is owing to the paroxysmal nature of atrial fibrillation and the variable symptoms of heart failure, which can be influenced by factors like medication adjustments, dietary changes, and the disease’s progressive course. In other words, the subject label is known, but the individual window labels are unknown. This is visually represented in Figure 2a, where the subject label is depicted by the blue/red colors and the unknown window labels are indicated by black dotted lines.

### Planned machine learning approach

**Figure 3:**
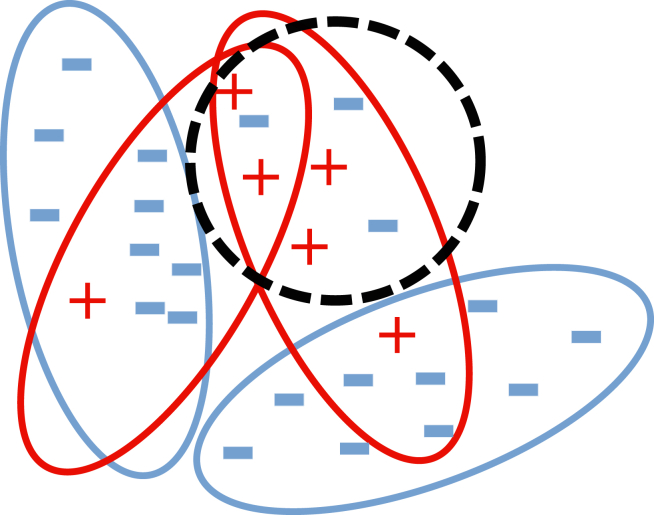
Illustration of multiple-instance learning. The red and blue lines indicate bags of heart disease patients and reference subjects. Even though the labels for each instance are not known, for the sake of this example, the plus and minus signs depict time windows where heart disease is present or absent, respectively. The decision boundary is depicted by a dotted circle, where instances within the circle are classified as heart disease, and instances outside the circle are classified as healthy.

Our planned machine learning approach is tailored to operate in this setting through a 2-stage process. To learn informative patterns/features directly from the input data, despite the lack of labeled windows, the first stage involves using self-supervised learning. A commonly used self-supervised learning technique involves compressing the input windows to a lower-dimensional representation and then reconstructing the original input from this compact representation, as depicted in Figure 2b.^7^^,^^8^ Instead of reconstruction, another technique is to forecast future time points of the input data.^9^ The second stage involves multiple-instance learning (MIL). MIL, depicted in Figure 3 and more elaborately explained in Box 1, is suitable for data where a single prediction is made collectively on a group of samples (known as a “bag”) instead of predicting on individual samples (known as “instances”). MIL techniques align well with our time series data, where during the training phase only 1 label related to the subject (bag label) is known while the individual labels of the compressed windows (instance labels) remain unknown. In the testing phase the primary objective is to predict 1 clinical outcome for each subject. The key concept in MIL is that each bag of instances is labeled as positive (heart disease) if it contains a certain amount of positive instances and negative (healthy) if it contains no positive instances (only negative instances). Although traditional MIL approaches often classify a bag as positive even with just 1 positive instance, we aim to minimize false-positives by setting a threshold on the number of positive instances required to label a bag as positive. This threshold will be determined through hyperparameter tuning. Thus, instead of learning a model that predicts the cardiovascular outcome of individual instances, we are learning a model that predicts the outcome of a bag of instances.

**Box 1. A simple multiple-instance learning example**

MIL is elaborated with an example in 3 steps.

**Initial setup** Under traditional supervised learning, each window must be annotated to train a machine learning model. However, in our MIL setting (Figure 3), only the bag label is known for the entire set of windows related to a subject. A bag label is considered negative if none of the subject’s individual samples are associated with heart disease, indicating that the subject is not affected by it. Conversely, a bag label is positive if a certain amount of the subject’s samples is linked to heart disease.

**Learning** The algorithm then learns a model based on the bag-level labels only. The goal of the MIL algorithm is to learn a model that can correctly predict the bag-level labels given the instances in each bag. By training on the bag-level labels, the MIL algorithm can capture patterns and relationships within the data that help identify the presence or absence of heart disease. Note that the decision boundary produced by the model in Figure 3 is not ideally suited for classifying individual windows, which is expected, as it did not use this information. However, if a sufficient number of windows are classified accurately, the correct bag label can still be predicted. This is accomplished during training by aggregating these accurate classifications using methods like majority voting, or by setting a threshold for the minimum number of positively predicted windows needed to assign a positive bag label.

**Prediction** Once the model is trained, it can predict the label of a new bag by examining the instances in the bag. If the model predicts that a certain percentage of instances in the bag are positive defined by the threshold, the bag is classified as positive (heart disease) and negative (healthy) otherwise. By analyzing the presence or absence of positive instances within the bag, the MIL algorithm can make predictions on a bag level, providing insights into the subject’s condition.

### Algorithm validation

**Figure 4:**
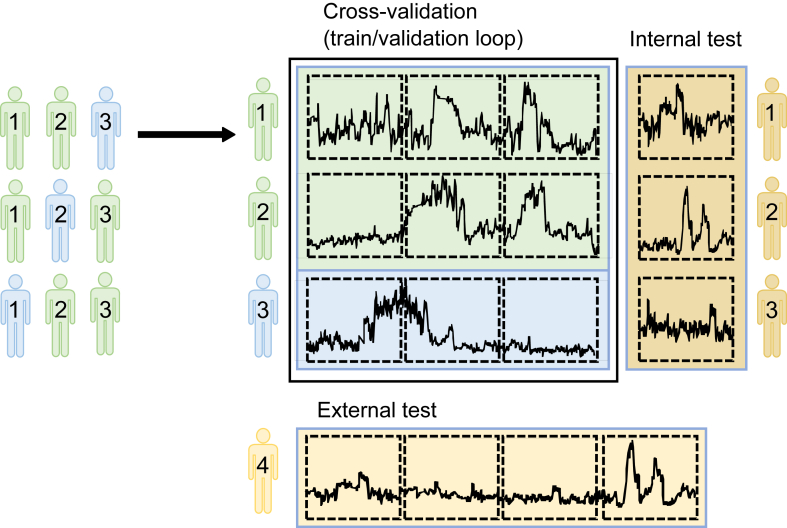
Our leave-p-subjects-out cross-validation strategy consists of the following: On the left, cross-validation folds are illustrated using 3 subjects with corresponding subject number. Green and blue represent training and validation subjects, respectively. On the right, a single fold is expanded and additionally illustrates the internal and external test sets. Each row corresponds to a subject, and the dotted squares within each row represent windows. The yellow external test block encompasses entire subjects and their corresponding data points that have not been encountered by the model. Meanwhile, the dark yellow internal test block consists of unobserved data points, representing the final 20% measurements of the time series from subjects already encountered during model development.

In our specific setting where the model encounters data from previously unseen subjects without any prior knowledge, we use leave-p-subjects-out cross-validation (LPSOCV). This approach, shown in Figure 4, ensures a more accurate reflection of real-world situations. LPSOCV involves multiple iterations, or folds, during which data from distinct subjects are used for training and validation purposes (ie, the model is validated on data from subjects that the model is not trained on), mitigating observation bias.

Machine learning models are sensitive to the distribution of classes. To mitigate potential bias owing to different class distributions in each fold, we incorporate stratification during the cross-validation process. This ensures that the ratio of nonarrhythmic, atrial fibrillation, and heart failure subjects remains approximately consistent and that the influence of inconsistent class distribution across different folds is minimized.

However, Fitbit time series data exhibit inter-subject variability resulting from individuals’ distinct physical attributes,^10^ making the development of a universally effective model for “new” subjects challenging. In order to examine the effect of inter-subject variability, we will assess the model on 2 distinct test sets. The first is an external test set that consists of subjects not previously encountered, randomly selected to make up 20% of the total subjects, with an equal number from each class. This allows us to evaluate the model’s generalization capabilities. The second test set is an internal one, encompassing the final 20% of data from subjects previously encountered by the model. This segment of data was excluded during the cross-validation phase and serves as a baseline, as it minimizes the influence of inter-subject variability, providing a reliable reference for comparison.

Parameters not directly learned by the machine learning model, such as window parameters, are termed hyperparameters. Since optimal values are typically unknown in advance, multiple options are examined during LPSOCV, and the best-performing one, with the best average performance over all folds, is chosen for the final model; a process known as hyperparameter tuning.

## Results

Preliminary findings are discussed in the following sections.

### Study characteristics

So far, 62 of the 200 envisioned subjects have been included and data from 22 subjects (15 healthy, 7 AF) have been extracted successfully from the data platform for preliminary analysis (Table 1).

**Table 1:** Characteristics of preliminary study participants as of May 2022

Characteristic	Ref	HF	AF
Participants, n	25	15	22
Age, y			
18–39	16	1	0
40–54	3	3	2
55–64	5	5	3
65+	1	6	17
Sex			
Male	12	12	15
Female	13	3	7
BMI			
18.5–24.9	14	3	7
25–29.9	6	6	8
30+	5	6	7
Diabetes			
Yes	0	5	4
No	25	10	18
Smoking			
Yes	0	6	5
No	25	9	17
Hypertension			
Yes	2	8	15
No	23	7	7
Device			
Charge 5	23	8	12
Inspire 2	2	7	10

AF = atrial fibrillation group; BMI = body mass index; HF = heart failure group; Ref = reference group.

### Data show large inter-subject variability

**Figure 5:**
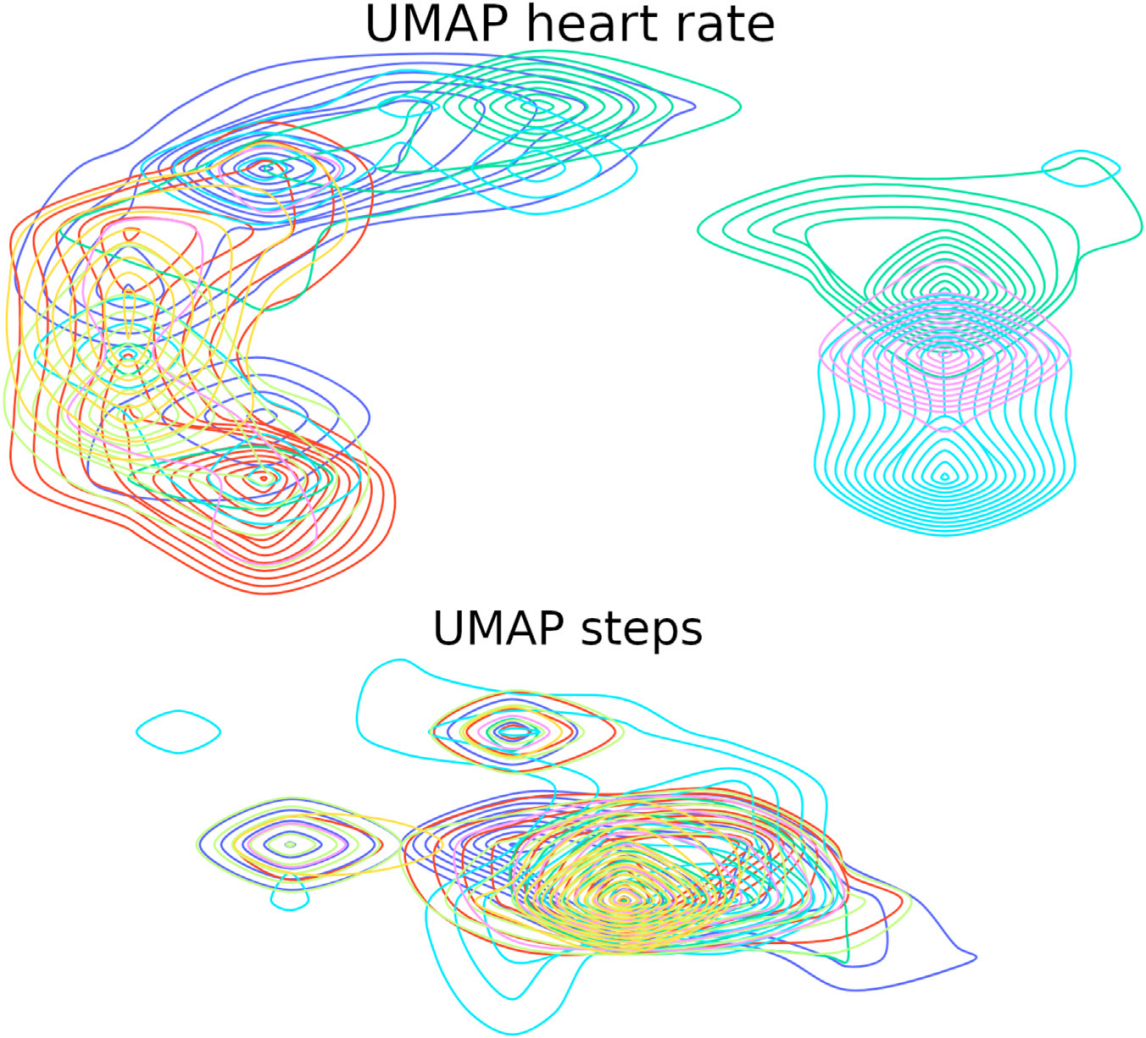
Visualization of heart rate windows (upper image) and step windows (lower image) of 6 subjects. Each window has data of 1 hour (720 time points for heart rate, 60 for steps, respectively). Each high-dimensional window is mapped to a 2-dimensional (2D) location using UMAP.^11^ The contour curves illustrate the distribution of the 2D UMAP samples for each subject, with each color representing 1 of the 6 subjects.

Nonoverlapping 1-hour windows are used to segment heart rate and step counter time series data from 6 subjects. To visualize this high-dimensional data, UMAP^11^ is employed to reduce the data to 2 dimensions while maintaining as much structure as possible. The resulting embedding is displayed in Figure 5, where the distribution of the 2-dimensional UMAP samples are illustrated per subject.

When there is little overlap between subjects, finding a shared pattern among them becomes challenging, making it difficult for a model to learn. As a result, the performance of a machine learning model could be impacted as, during testing, a subject can significantly deviate from the subjects on which the model was trained.

### Heart rate peak alignment in acceleration-deceleration curves indicate difference between 7 AF patients and 15 healthy controls

**Figure 6:**
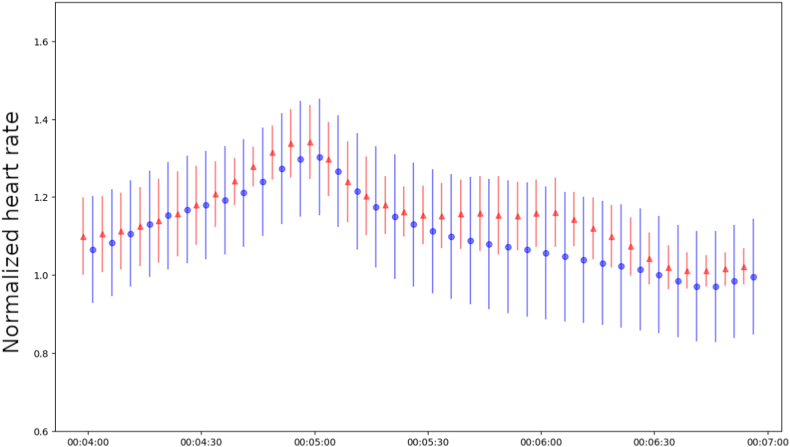
Acceleration-deceleration curves during light activity. Red and blue represent data from subjects with persistent atrial fibrillation (n=7) and no heart disease (n=15), respectively. The mean and standard deviation are shown per time point (5 second intervals) for both groups.

Next, we explored the heart rate recovery curves after activity (acceleration-deceleration curves).^12^ First a peak is detected, whereafter the start (onset) and end (recovery) points associated to that peak are determined by the minimum heart rate value 5 minutes before and 15 minutes after the peak. The curves are preprocessed by aligning the peaks on the time axis. Additionally, for every subject, the amplitude of the curves is rescaled by the average peak value across all curves for that individual. Figure 6 shows the curves for light activity, defined by a maximum of 20 steps in the 5 minutes preceding the peak and fewer than 10 steps in the 15 minutes after the peak. There are 2 noticeable differences in heart rate patterns between persistent AF patients (in red) and healthy participants (in blue). The standard deviation for AF patients is considerably smaller than that of healthy individuals, and their heart rate recovery is slower, as observed at the 6-minute mark. These distinctions could potentially serve as clinical indicators for atrial fibrillation.

### MIL can detect healthy cardiovascular outcomes

The peak aligned acceleration-deceleration curves are concatenated with their corresponding step counter data and grouped per week to form bags. The MILES (Multiple-Instance Learning via Embedded Instance Selection) model is then used to classify every week as healthy or AF. The results in Table 2 show that even though the sensitivity is low, the specificity is decent. This shows potential in avoiding unnecessary visits to a cardiologist for patients who have symptoms that are wrongly suspected to be related to heart problems.

**Table 2:** Confusion matrix of per-week healthy vs atrial fibrillation classification of the MILES model with peak aligned curves concatenated with step counter data, with true and predicted labels shown vertically and horizontally, respectively

True/Predicted label	AF	Healthy
AF	11	14
Healthy	7	33

AF = atrial fibrillation.

### Step counter and heart rate are correlated with a time delay

**Figure 7:**
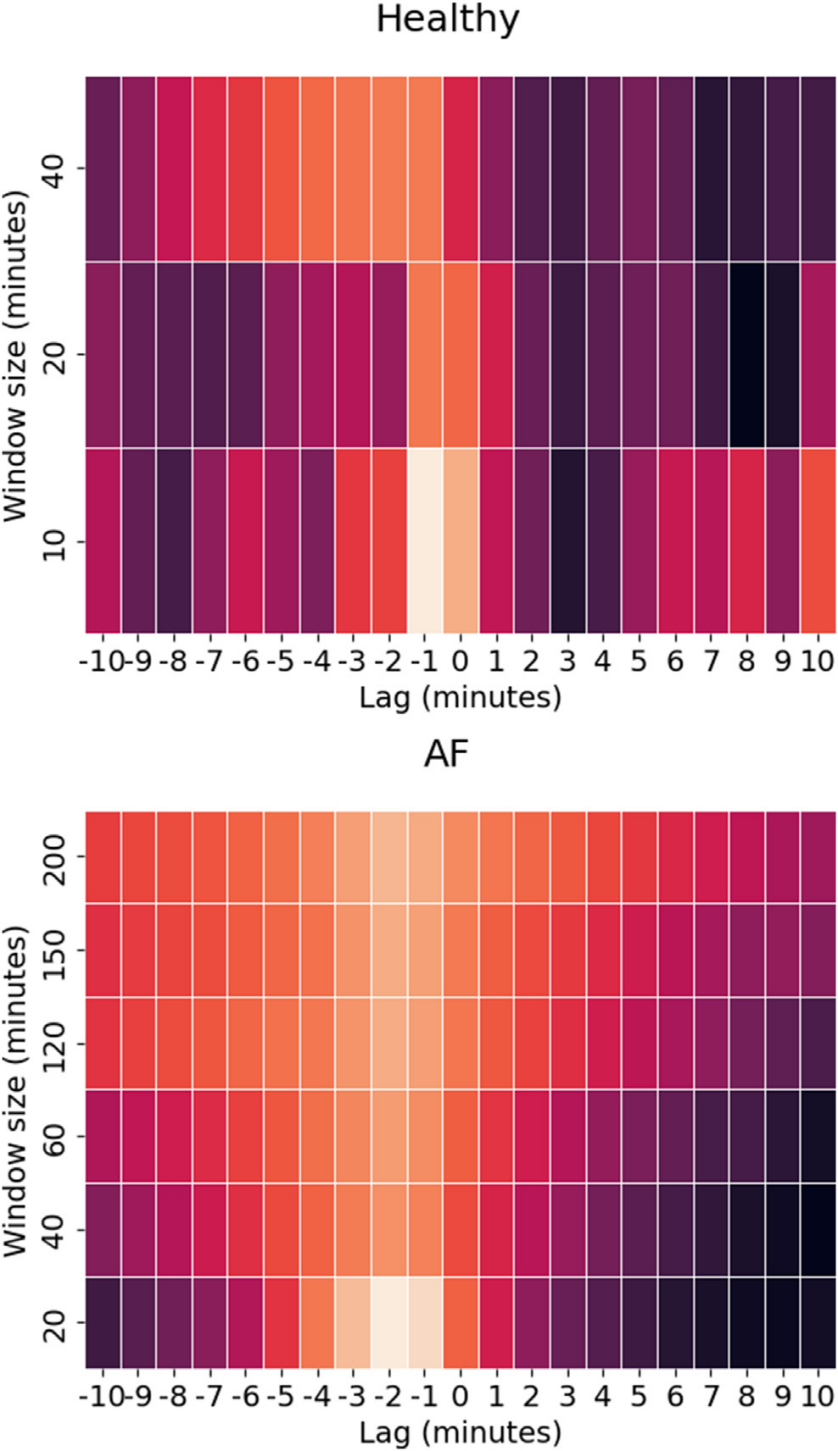
Cross-correlation matrices between windowed heart rate data and number of steps for healthy and the persistent atrial fibrillation (AF) group. Rows are window sizes and columns lag between heart rate and step counter.

Next, we examined whether the cross-correlation function between the heart rate window and its corresponding step counter window is indicative of heart disease. To calculate the correlation, we consider varying window sizes and time differences (lags) between heart rate and steps. The computed cross-correlation matrix for the healthy group, along with the AF and HF patient groups, as shown in Figure 7, shows that the heart rate is correlated with the step counter with 1-minute delay.

## Discussion

By building a suitable infrastructure with Cloud technology, big data acquired in the study is used to develop data-efficient models using methods from multiple instance and self-supervised learning.

We aim to examine the influence of inter-subject variability on predicting cardiovascular disease and will explore potential methods to mitigate these variabilities.^13^^,^^14^ We expect that patterns indicative of cardiovascular disease become apparent within a timeframe of minutes, hours, or more, considering that consumer-grade wearables have a slower sampling rate compared to the gold standard. We have shown 1 example of such a pattern: the acceleration-deceleration curve. Preselecting windows based on such patterns furthermore mitigates searching through substantial amounts of data that may not provide much information about cardiovascular disease. Inspecting the cross-correlation for several combinations of window size and lag show a different profile for healthy and AF group individuals, showing that it is meaningful to analyze step counter and heart rate together. Big data for heart disease detection requires substantial labeling efforts from physicians.

Using self-supervised learning and MIL, a model can be trained with much fewer labels. Our findings demonstrate this by employing MILES to achieve high specificity, which can aid in ruling out heart disease in individuals experiencing symptoms similar to heart disease but without the condition (ie, false-positives).

## Conclusion

The ongoing ME-TIME study is a longitudinal observational study that uses machine learning with time series data from consumer-grade smartwatches to detect atrial fibrillation and heart failure. This will contribute to cost-effective cardiovascular monitoring of outpatients, thereby reducing exacerbation of cardiovascular disease and effectively increasing capacity of global cardiovascular healthcare.

## Declaration of generative AI and AI-assisted technologies in the writing process

During the preparation of this work the first author used chatGPT in order to correct spelling and grammar and to improve sentence clarity. After using this tool/service, the authors reviewed and edited the content as needed and take full responsibility for the content of the publication.
